# TMEM116 is required for lung cancer cell motility and metastasis through PDK1 signaling pathway

**DOI:** 10.1038/s41419-021-04369-1

**Published:** 2021-11-16

**Authors:** Suhong Zhang, Haiting Dai, Wenya Li, Runming Wang, Hanyu Wu, Ming Shen, Ye Hu, Lixin Xie, Yiming Xing

**Affiliations:** 1 State Key Laboratory for Agrobiotechnology, College of Biological Sciences, China Agricultural University, Beijing, P. R. China; 2 Translational Medicine Laboratory, Chinese PLA General Hospital, Beijing, P. R. China; 3 College of Pulmonary and Critical Care Medicine, Chinese PLA General Hospital, Beijing, P. R. China

**Keywords:** Non-small-cell lung cancer, Non-small-cell lung cancer

## Abstract

Transmembrane protein (TMEM) is a family of protein that spans cytoplasmic membranes and allows cell–cell and cell–environment communication. Dysregulation of TMEMs has been observed in multiple cancers. However, little is known about TMEM116 in cancer development. In this study, we demonstrate that TMEM116 is highly expressed in non-small-cell lung cancer (NSCLC) tissues and cell lines. Inactivation of TMEM116 reduced cell proliferation, migration and invasiveness of human cancer cells and suppressed A549 induced tumor metastasis in mouse lungs. In addition, TMEM116 deficiency inhibited PDK1-AKT-FOXO3A signaling pathway, resulting in accumulation of TAp63, while activation of PDK1 largely reversed the TMEM116 deficiency induced defects in cancer cell motility, migration and invasive. Together, these results demonstrate that TMEM116 is a critical integrator of oncogenic signaling in cancer metastasis.

## Introduction

Membrane proteins represent a large population of cellular proteins and a lot of them play a fundamental role during cancer development by transmitting information between the extracellular environment and the cytoplasmic proteins. However, many transmembrane proteins are poorly described and are grouped in the transmembrane protein (TMEM) family. The term TMEM reflects not only the nature of the protein but also the lack of information about their possible structures, functions and mechanisms [1]. Many studies showed that TMEM expression could be up- or down-regulated in tumor tissues, such as in lymphomas (TMEM176) [2], colorectal cancer (TMEM25) [3], hepatic cancer (TMEM7) [4], and lung cancer (TMEM48) [5]. Some of them are used as prognostic biomarkers [6]. Functionally, several TMEM family members have been described as tumor suppressors while others as oncogenes [7]. Since a number of TMEMs have been implicated in cancer development and in drug resistance, the TMEM family has drawn a lot of interest in cancer research.

Lung cancer is the most common cause of mortality and accounts for almost 18.4% of deaths due to cancer worldwide [8]. Previous studies showed that aberrant activation of oncogenic pathways and inactivation of tumor-suppressor pathways lead to malignant expansion of cancer cells in the lung. Subsequently, irreversible alterations in the cellular functions give rise to dysplasia and clonal patches [9]. The majority of cancer-associated deaths are related to secondary tumor formation. This multistep process involves the migration of cancer cells from primary tumor sites to distant organs. As the invasion and metastasis of cancer cells are keys to patient survival, a better understanding of the precise mechanics of cancer cell migration opens perspectives for better cancer patient care.

Although the compositions of lung cells are different, distinct lung cancer subtypes share some similarities in pathogenesis, histopathological and oncogenesis. For example, abnormalities in phosphatidyl inositol 3-kinases (PI3K) signaling are found in variety of lung cancers. PI3Ks are a family of lipid kinases that regulate multiple cellular functions including cell proliferation, survival, differentiation, adhesion, motility and invasion [10]. PI3Ks are divided into three subclasses on the basis of structure, regulation, and lipid substrate specificity. Class I PI3Ks are heterodimeric proteins formed of a p110 catalytic subunit (p110α, p110β, or p110γ, encoded by *PIK3CA*, *PIK3CB*, and *PIK3CD*, respectively) and a p85 regulatory subunit, which is primarily involved in the pathogenesis of human cancer [11]. The oncogenic hotspot mutations of *PIK3CA* are found within the p110α helical domain or the kinase domain [12]. Active PI3Ks phosphorylate phosphatidyl inositol 4,5-bisphosphate (PIP2) to form phosphatidyl inositol 3,4,5-triphosphate PIP3. PIP3 acts as a docking site for pleckstrin homology (PH)-containing proteins, including 3-phosphoinositide dependent protein kinase 1 (PDK1) and the protein kinase B (AKT) [13].

PDK1 phosphorylates AKT [14], and many other AGC kinases [15]. Aberrant activation of these PDK1 downstream targets has been proved to play key roles in uncontrolled cell replication, apoptosis escape, invasion and dissemination, metastasis, metastasis, metabolic reprogramming and abnormal angiogenesis [16]. Many human tumors show high PDK1 expression due to gene amplification. Interestingly, increased PDK1 gene copy number is frequently associated with other genetic mutations in PI3K/Akt pathway [17]. Moreover, higher PDK1 expression is associated with advanced tumor stage (positive lymph node metastasis or high histological grade) and shorter overall survival [15]. As inhibition, silencing or gene ablation of PDK1 in experimental models have highlighted its suitability as a potential therapeutic target in breast [15], pancreatic cancers [18], and melanoma [19], understanding the precise mechanics of how PDK1 drives lung cancer development is of significant interest.

AKT is common downstream effector of PI3K signaling pathway and considered as a master regulator of tumor cell proliferation, survival, and motility [20, 21]. Activated Akt is capable of phosphorylating various substrates, including a subset of fork-head transcription factor (FOXO), mammalian target of rapamycin (mTOR), IKK, and GSK3β [22]. FKHRL1 (FOXO3A), a member of FOXO family, regulates expression of specific sets of genes involved in apoptosis, stem cell self-renewal, stress resistance, and longevity [22]. Down-regulation of FOXO3A is associated with cancer development [13]. p63 is a member of p53 family that is involved in a wide range of biological processes. Due to an alternative transcription start site, the p63 is expressed as either TAp63 isoforms or ΔNp63 isoforms with or without N-terminal transactivation (TA) domain homologous to that of p53. Both TAp63 and ΔNp63 play unique and overlapping roles in cancer development. Deletion of TAp63 in mice caused highly metastatic carcinomas and sarcomas [23]. As a direct transcriptional target of FOXO3A, ΔNp63α is critical in p110α^H1047R^-mediated cell motility cancer metastasis. Moreover, several signaling pathways, such as Ras and Her2, promote cancer cell motility and tumor metastasis by targeting and repressing ΔNp63α expression [14].

TMEM116 is a member of TMEM family with unknown functions. In this study, we detected that TMEM116 expression is robustly increased in human lung cancer clinical samples and mouse lung cancer models. Increased level of TMEM116 was observed in human lung cancer cell lines as compared to normal human bronchial epithelial cells. Targeting TMEM116 by CRISPR-Cas9 in A549 cells significantly reduced cell migration in vitro and tumor metastasis in vivo. Further analysis revealed the latter functions of TMEM116 is mediated through PDK1/AKT/FOXO3A/TAp63 signaling. Current study is the first to demonstrate the role of TMEM116 in lung oncogenesis and suggest that TMEM116 may be a potential therapeutic target of lung cancer.

## Materials and methods

### Cell lines and cell culture

Human pulmonary carcinoma H441, A549, H1299 cells and bronchial epithelial 16HBE cells were purchased from China Center for Type Culture Collection, China. The cells were cultured in RPMI-1640 or DMEM medium supplemented with 10% fetal bovine serum (Gibco, USA), 100 U/mL penicillin and 100 μg/mL streptomycin (Gibco, USA) at 37 °C in humidified air containing 5% CO_2_.

### NSCLC tissue samples

Human lung adenocarcinoma or squamous carcinoma specimens were collected and studied according to the protocol approved by the General Hospital of the People’s Liberation Army (S2016-057-02).

### Animal models

All animals were housed in pathogen-free conditions according to the protocol approved by Beijing Association on Laboratory Animal Care (Beijing, China) and all animal studies were conducted according to protocols approved by China Agricultural University (SKLAB-2016-18). *Dermo1-Cre* and *ROSA*^*mTmG*^ mice were gifts from Dr. Parviz Minoo (University of Southern California, USA). 5–6 weeks old female A/J mice were exposed to BaP (100 mg/kg body weight twice per week for 5 weeks) in corn oil via oral gavage [24]. All treated animals sacrificed in sixth month after BaP treatment (six mice per experimental group). Both A/J and BALB/c nude mice were purchased from Charles River/Vitalriver China. To establish subcutaneous tumorigenesis model, 5.0×10^6^ A549 cells were injected into the flanks of 6 weeks old female BALB/c nude mice (six mice per experimental group). Tumor size was measured and all mice were sacrificed after 7 weeks. For the metastasis analysis, 3.0 × 10^6^ cells were injected into the tail vein of 6 weeks old female BALB/c nude mice (six mice per group).

### RT-PCR and qPCR

Total RNA was extracted from lungs or cells by Qiagen RNeasy Mini Kit (QIAGEN, Germany) according to the manufacturer’s protocol. RNA was reverse-transcribed into cDNAs using M-MLV Reverse Transcriptase Kit (Promega, USA). Quantification of selected genes by real-time PCR was performed using LightCycler (Roche Diagnostics, Germany). Primers used for RT-PCR and qPCR were designed by Primer3 software. GAPDH expression was used as an endogenous control to normalize target gene expression. The primers used were listed in Supplementary Table 1.

### Western blot

Cells or lung tissues were harvested and frozen in liquid nitrogen. Protein extracts were prepared by using RIPA reagent kit (Beyotime, China) containing 1 mM PMSF (Beyotime, China) and PhosSTOP EASY pack (Roche, Switzerland). Equal amounts of protein were separated by SDS/PAGE gels, transferred to Immobilon-P transfer membranes, and hybridized to an appropriate primary antibody and HRP-conjugated secondary antibody for subsequent detection by enhanced chemiluminescence (Millipore). The intensity of bands was analyzed by Image J. Primary antibodies used in this study were listed in Supplemental Table 2.

### Immunohistochemistry

Lungs were fixed in 4% paraformaldehyde in phosphate buffed saline (PBS; pH7.0) and processed into serial paraffin sections (4 µm) using standard procedures. Sections were incubated with primary antibodies listed in Supplementary Table 2. Haematoxylin-eosin (H&E) staining was performed for morphological examination. Size of the scale bar for each experiment is indicated in the figure legends. To quantify the number of Vimentin and N-cadherin positive cells, percentage of Vimentin and N-cadherin positive cells in total cells were counted on control and tumor area.

### Cell transfection experiments

SgRNA expression vectors against human TMEM116 were obtained from Cyagen Biosciences Inc and subcloned into pCRISPR-W2 vector. 2 μg of pCRISPR-W2-TMEM116 plasmids were prepared and transfected into A549 cell lines by using Lipofectamine 2000 kit (Invitrogen, USA). After 48 h, the cells were cultured in 800 μg/ml G418 for 1 week to select the positive cells. Selected mutant clones were grown on 96-well plates for further examination. The sequences of TMEM116 sgRNAs were as follows: sgRNA1: 5′-ACGGGTGCGCTTCTACCCAG-3′; sgRNA2: 5′-CATA AAGCTGACTAAGCCAC-3′.

### Wound-healing assay, migration and invasion assay

For wound-healing assay, cells were grown to 90% confluency in growth media, and then wounded with a plastic pipette tip. Cells were then washed twice with PBS and incubated in media containing 1% serum at 37 °C in a humidified incubator under 5% CO_2_. Immediately after a scratch was created, markings were made adjacent to the scratch as a reference for camera positioning. Photographs were taken at the initial and 48 h later by phase-contrast microscope (Nikon Eclipse Ti-S/L 100). Then percentage of migrating area in initial scratch area was quantified by ImageJ. Over 30 non-overlapping fields in each sample were analyzed at each time point, and at least three independent experiments were performed. Transwell assays for migration were performed in transwell inserts with a 6.5-mm, 8.0-μm-pore polycarbonate membrane, or Matrigel coated inserts for invasion assays (BD Biosciences). Briefly, cells were suspended in serum-free media and seeded into the inner chamber. The outer chamber contained complete growth media. Cells were incubated for 24 h and then non-migrating or non-invading cells on the inside of the membrane were carefully removed with a cotton swab, while migrating/invading cells on the outside of the membrane were fixed and stained with 0.5% Crystal violet in 70% ethanol, photographed under a light microscope. At least five random fields (200×) were analyzed for the number of invading cells [25].

### Cell morphology and colony formation assays

For assessing cell morphology, cells were seeded in six-well plates at low confluency (500 cells/well) and grow for 2 weeks, then fixed with methanol and stained with 0.1% crystal violet in 70% ethanol. Cell colonies were counted and photographed to assess cell morphology under a light microscope.

### Cell viability assays

Cell viability was assessed using CCK8 assay kit (Beyotime, China) according to the manufacturer’s instructions. The absorbance was determined at 450 nm wave length.

### Statistical analysis

Statistical analyses were performed with SPSS 16.0 software. Statistical significance between two groups was determined using an unpaired two-tailed Student’s *t* test. Data are presented as mean ± SD (standard deviation) as indicated in the figure legends. *P*-values were considered statistically significant at *P* < 0.05.

## Result

### TMEM116 is highly expressed in mouse lung epithelial cells

**Fig. 1:**
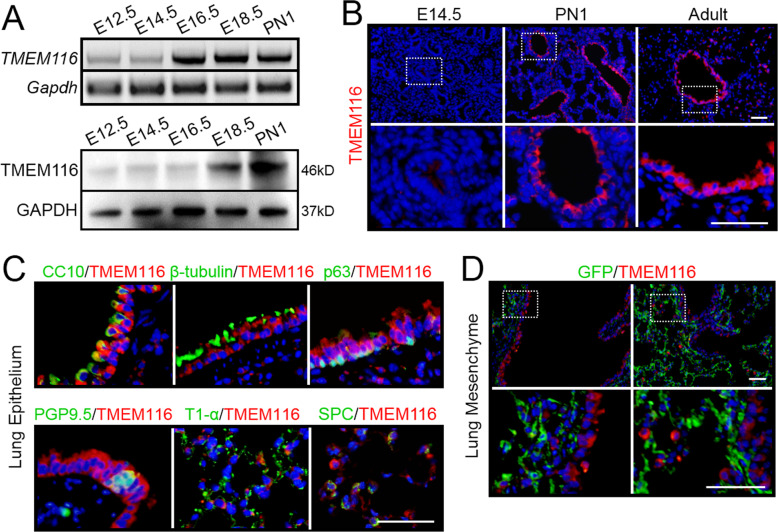
The expression pattern of TMEM116 in mouse lung. **A** The expression level of *TMEM116* in mouse embryonic lungs were examined by RT-PCR and western blot analyses. **B** Immuno-fluorescence (IF) staining analysis showed TMEM116 expression in E14.5, PN1 and adult mouse lungs. **C** Double IF staining analysis of TMEM116 and p63, β-tubulin, CC10, PGP9.5, T1-α, and Spc respectively. **D** IF staining analysis of TMEM116 and GFP in *Dermo1-cre; ROSA*^*mTmG*^ mice. Representative images from three independent experiments are shown above. Scale bar: 50 μm.

TMEM116 is a member of TMEM protein family with unknown functions. By reverse transcription PCR and western blot analysis, we found that *TMEM116* was highly expressed in mouse kidney, lung and heart (Supplementary Fig. 1). During the mouse lung embryonic development, expression of *TMEM116* dramatically increased since E16.5 and remained high until postnatal stage (Fig. 1A). By immunohistochemistry analysis, we found that *TMEM116* expression was barely detectable in E14.5 lungs, whereas the level of TMEM116 increased in airway epithelium and alveolus at postnatal day 1 (PN1) and adult stages (Fig. 1B). Double staining with antibodies against specific cell type markers revealed that TMEM116 positive cells include airway Club cells (CC10 positive cells), ciliated cells (β-tubulin positive cells), basal cells (p63 positive cells), neuroendocrine cells (PGP9.5 positive cells), alveolar type I (T1-α positive cells), and type II (SPC positive cells) (Fig. 1C).

To clarify whether *TMEM116* was expressed in lung mesenchymal cells, we generated *Dermo1-cre; Rosa26*^*mTmG*^ mice. Dermo1 is a general marker of lung mesenchymal cells. In *Dermo1-cre; Rosa26*^*mTmG*^ mouse lung, the mesenchymal cells were labeled by GFP. Double staining of GFP and TMEM116 showed no overlap indicating that the mesenchymal cells do not express *TMEM116* in mouse lung (Fig. 1D).

### TMEM116 expression is promoted in lung cancer

**Fig. 2:**
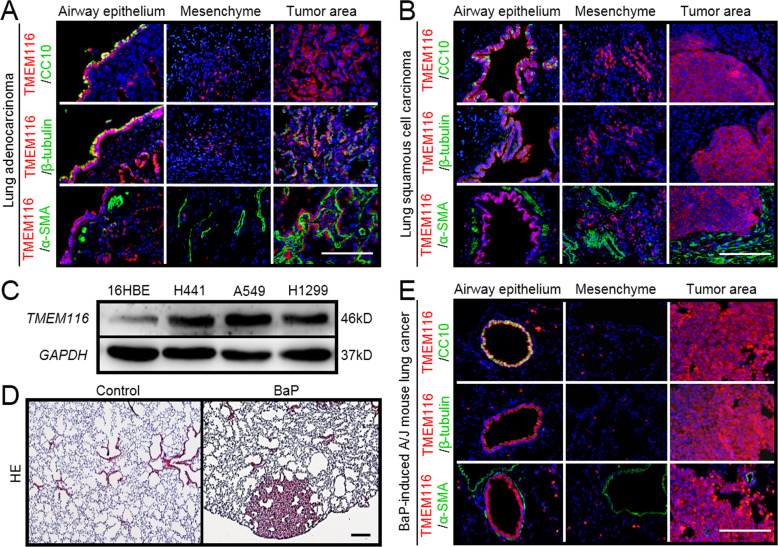
TMEM116 expression is promoted in lung cancer. **A** Double IF staining analysis of TMEM116 and CC10, β-tubulin, and αSMA respectively in human LUAD tissues and adjacent tissues. **B** Double IF staining of TMEM116 and CC10, β-tubulin, and αSMA respectively in human LUSC tissues and adjacent tissues. **C** The expression of TMEM116 in the 16HBE, A549, H1299, H441 cells by western blot analysis. **D** H&E staining analysis of BaP-induced A/J mouse lung tissue. **E** Double IF staining analysis of TMEM116 and CC10, β-tubulin, and αSMA respectively in A/J mouse lung cancer tissues and normal tissues adjacent to carcinoma. Representative images from three independent experiments are shown above. Scale bar: 200 μm.

In previous studies, several TMEM family members have been reported to be up- or down-regulated in cancers. To investigate whether TMEM116 expression was altered in lung cancer, we examined its expression in the clinical samples of two main subtypes of non-small-cell lung cancer (NSCLC), lung adenocarcinoma (LUAD) and lung squamous cell carcinomas (LUSC). As show in Fig. 2A, B, expression of *TMEM116* was strongly activated in tumor areas in both LUAD and LUSC lungs. Interestingly, TMEM116 positive cells in tumor areas were not labeled by either airway epithelial cell markers (CC10 and β-tubulin) or mesenchymal cell marker (α-SMA). Whereas, in non-tumor areas TMEM116 was expressed in airway and alveolar epithelial cells (Fig. 2A, B). Western blot analysis result confirmed that *TMEM116* was highly expressed in human cancer cell lines A549 and H1299, as compared to non-tumor human bronchial epithelial cell line 16HBE (Fig. 2C).

To test whether *TMEM116* was also increased in mouse lung cancer. A/J mice were treated with 50–100 mg/kg benzo(a)pyrene (BaP) or vehicle control (corn oil). All BaP-treated animals developed lung cancer within 6 months and were analyzed by Hematoxylin and eosin (H&E) staining (Fig. 2, Panel D). In consistent with findings in human lung cancer, *TMEM116* was highly expressed in mouse tumor cells which had lost the characteristics of the airway epithelial cells (Fig. 2, Panel E). To test whether the TMEM116 positive cells underwent epithelial-mesenchymal transition (EMT) in tumor tissues, we conducted immunostaining with antibody against Vimentin and N-Cadherin. As shown in Supplementary Fig. 2, TMEM116 positive cells were negative for the latter mesenchymal cell markers.

By using GEPIA dataset, the expression of *TMEM116* in NSCLC and normal lung tissues was analyzed. As present in Supplementary Fig. 3A, B, *TMEM116* expression in both LUAD and LUSC were higher than that in normal lung tissues. Analysis of *TMEM116* expression in different stage of LUAD and LUSC showed no significant difference (Supplementary Fig. 3C, D). Then, Kaplan–Meier Plotter database was analyzed to reveal the association between *TMEM116* expression and overall survival (OS) time in patients with LUAD or LUSC (Supplementary Fig. 3E, F). The result indicated that higher expression of *TMEM116* correlated to shorter OS of LUAD, but not LUSC.

### TMEM116 deficiency inhibits scattered cell growth, clone formation, cell proliferation, invasion and migration

To investigate the role of TMEM116 in lung tumorigenesis, we designed sgRNA targeting *TMEM116* in human lung carcinoma cell line A549 (Supplementary Fig. 4A). Based on DNA sequencing result, we selected two *TMEM116* mutant cell lines (named *TMEM116*^*KD*^*-*1 and *TMEM116*^*KD*^-2, Supplemental Fig. 4B) for future experiments. Western blot analyses showed that the expression levels of TMEM116 were significantly reduced in these mutant cell lines (Supplementary Fig. 4C).

**Fig. 3:**
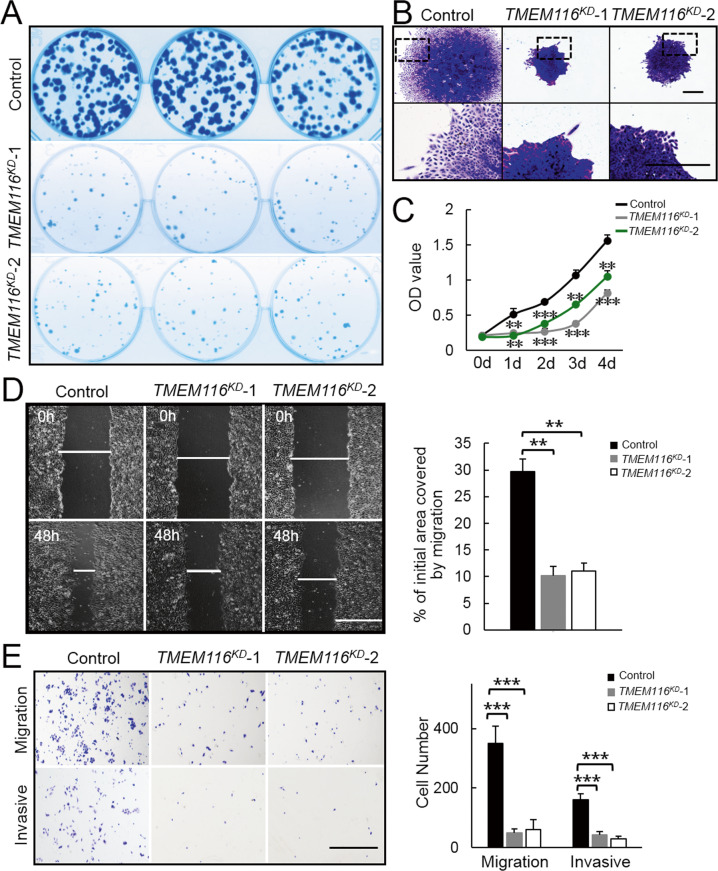
TMEM116 deficiency inhibits scattered cell growth, clone formation, cell proliferation, invasion and migration. **A** *TMEM116*^*KD*^ and control cells were subjected to colony formation assays. **B** *TMEM116*^*KD*^ and control cells were subjected to colony morphology assays. Scale bar: 500 μm. **C** *TMEM116*^*KD*^ and control cells were subjected to CCK-8 proliferation assay. **D** *TMEM116*^*KD*^ and control cells were subjected to wound-healing assay. Scale bar: 1000μm. Representative images from over 30 non-overlapping fields at each time point are shown. **E** *TMEM116*^*KD*^ and control cells were subjected to Transwell migration and invasion assays. Scale bar: 1000 μm. The bars represent the mean ± SD. *P < 0.05, **P < 0.01, ***P < 0.001. Representative images from three independent experiments are shown above.

We therefore examined the effect of TMEM116 deficiency on cell morphology, cell migration, and invasion in TMEM116-knockdown (*TMEM116*^*KD*^) A549 cells. After 2 weeks culture, *TMEM116*^*KD*^ cells formed much less and highly compact colonies with clear edges, whereas control A549 cells formed more colonies and showed scattered growth pattern with spindle-like cell shape (Fig. 3A, B). CCK8 assay demonstrated that the proliferation and viability were decreased in *TMEM116*^*KD*^ cells vs control cells (Fig. 3C). In addition, knockdown *TMEM116* in A549 cells dramatically inhibited cell migration and cell invasion as shown in wound-healing assay (Fig. 3D) and transwell assays (Fig. 3E).

### TMEM116 deficiency suppresses tumor metastasis

**Fig. 4:**
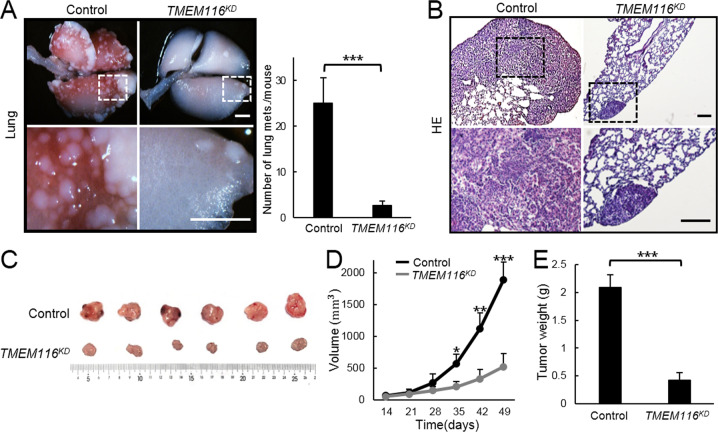
TMEM116 deficiency suppresses tumor growth and metastasis in vivo. **A** After tail vein injection by *TMEM116*^*KD*^ or control cells, metastatic nodules on lung surface were analyzed. Scale bar: 2 mm. **B** The xenografts tumorigenesis were analyzed by H&E staining. Scale bar: 200 μm. After subcutaneous injection into the flanks, tumor size (**C**), tumor volume (**D**), and tumor weight (**E**) were measured and analyzed. *P < 0.05, **P < 0.01, ***P < 0.001. Representative images from three independent experiments are shown above.

To further investigate the pathological role of TMEM116 in tumorigenesis and tumor metastasis in vivo, we established xenograft models by injecting stable *TMEM116*^*KD*^ A549 cells or control cells (*n* = 6 per group) into BALB/c nude mice. After tail vein injection, mice bearing control A549 cells formed multiple metastasis nodules on the lung surface, whereas, lungs of *TMEM116*^*KD*^ A549 injected mice exhibited significantly reduced number of metastasis nodules (Fig. 4A). This was further confirmed histologically by H&E staining of lung sections (Fig. 4B). In addition, after subcutaneous injection into the flanks, the xenografts tumorigenesis derived from *TMEM116*^*KD*^ A549 cells were much smaller and lighter than that from control cells (Fig. 4C–E). Collectively, these data strongly demonstrate the importance of TMEM116 in tumor metastasis in vivo.

### Effects of TMEM116 on cell migration/invasion is through PDK1/AKT/FOXO3A/TAp63 signaling pathway

**Fig. 5:**
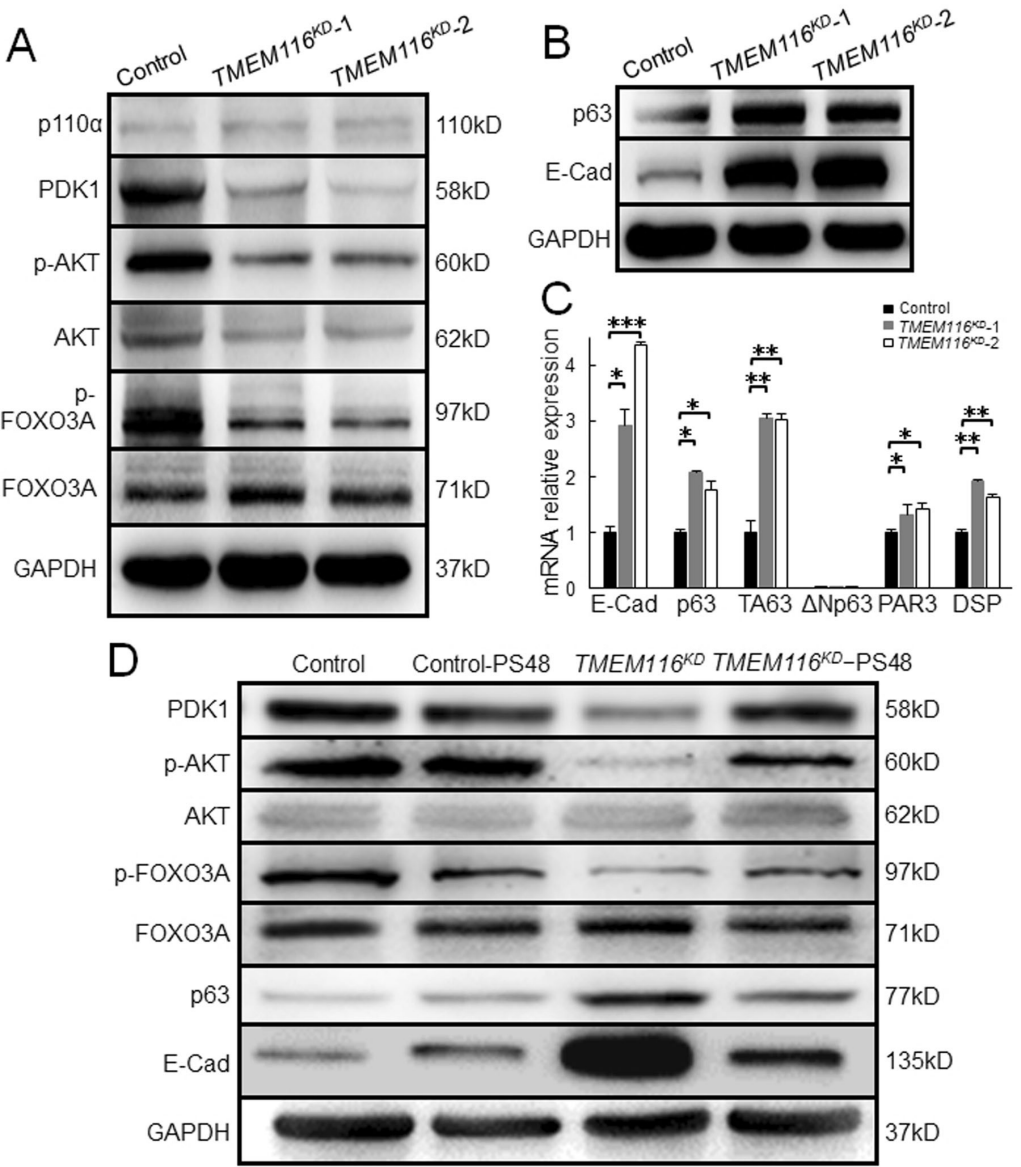
Effects of TMEM116 on cell migration/invasion is through PDK1/AKT/FOXO3A/TAp63 signaling pathway. **A** Western blot analysis of p110α, PDK1, p-AKT, AKT, p-FOXO3A, FOXO3A in control and *TMEM116*^*KD*^ A549 cells. **B** *TMEM116*^*KD*^ and control cells expressing p63 and E-cadherin were subjected to western blot analyses. **C** Real-time PCR analysis *of E-Cad*, *p63*, *TAp63*, *ΔNp63*, *DSP* and *PAR3* in control and *TMEM116*^*KD*^ cells. **D** *TMEM116*^*KD*^, *TMEM116*^*KD*^-PS48, control-PS48 and control cells expressing PDK1, p-AKT, AKT, p-FOXO3A, FOXO3A, p63, E-Cad were subjected to western blot analyses. Data represent the mean ± SD. *P < 0.05, **P < 0.01, ***P < 0.001. Representative images from three independent experiments are shown above.

PI3K is a classical signaling closely related to tumor diseases [26]. To determine whether PI3K/AKT/FOXO3A signaling pathway mediates the effects of TMEM116 in growth, colony formation, migration and invasion of A549 cells, we first determined expression of p110α, PDK1, AKT and FOXO3A in control and *TMEM116*^*KD*^ A549 cells. The results showed that TMEM116 deficiency reduced protein level of PDK1 as well as phosphorylation of AKT and FOXO3A, but not p110α (Fig. 5A; Supplementary Fig. 5A, B).

TP63 gene encodes multiple isotypes including TAp63 and ΔNp63α. Previous studies proved that suppression of ΔNp63α expression via AKT-FOXO3A signaling resulted in increased cell motility and tumor metastasis [13, 27]. We therefore examined whether TMEM116 deficiency altered expression of p63 and its downstream targets. By western blot, we found that p63 was indeed increased in *TMEM116*^*KD*^ A549 cell. Consistently, transcription of p63 targets such as Desmoplakin (*DSP*), par-3 family cell polarity regulator (*PARD3*), and E- cadherin 1 (*E-Cad*) [28] were also increased in the mutant cells (Fig. 5B, C; Supplementary Fig. 5C). To identify which isoform of p63 was involved in the effects of TMEM116 on A549 cells, the TAp63 and ΔNp63 expression in *TMEM116*^*KD*^ A549 cells were investigated. Interestingly, the expression of *TAp63* was greatly increased in *TMEM116*^*KD*^ A549 cells, whereas the expression of *ΔNp63* was too low to be detected in both *TMEM116*^*KD*^ and control A549 cells. This result suggested that TAp63 played key roles in TMEM116-mediated cell migration and invasion in A549 cells.

To investigate whether TMEM116 deficiency affected other signaling pathways related to oncogenesis, by western blot and reverse transcription PCR analyses, the expression of NFκB, PTEN, KRAS, RALA, RAF1, phosphorylation of mTOR and ERK was detected. The results showed no significant difference between *TMEM116*^*KD*^ and control A549 cells (Supplementary Fig. 6).

### Activation of PDK1 largely restores the proliferation, clone formation, migration and invasion of *TMEM116*^*KD*^ cells

To determine whether activation of PDK1 rescues the effects of TMEM116 deficiency on AKT/FOXO3A/p63 pathway, we treated *TMEM116*^*KD*^ A549 cells with PDK1 activator, PS48 [29]. As shown in Fig. 5, alterations of PDK1, p-AKT, p-FOXO3A, p63, and E-cadherin caused by TMEM116 deficiency were corrected by PS48 treatment in *TMEM116*^*KD*^ A549 cells (Fig. 5D; Supplementary Fig. 7).

**Fig. 6:**
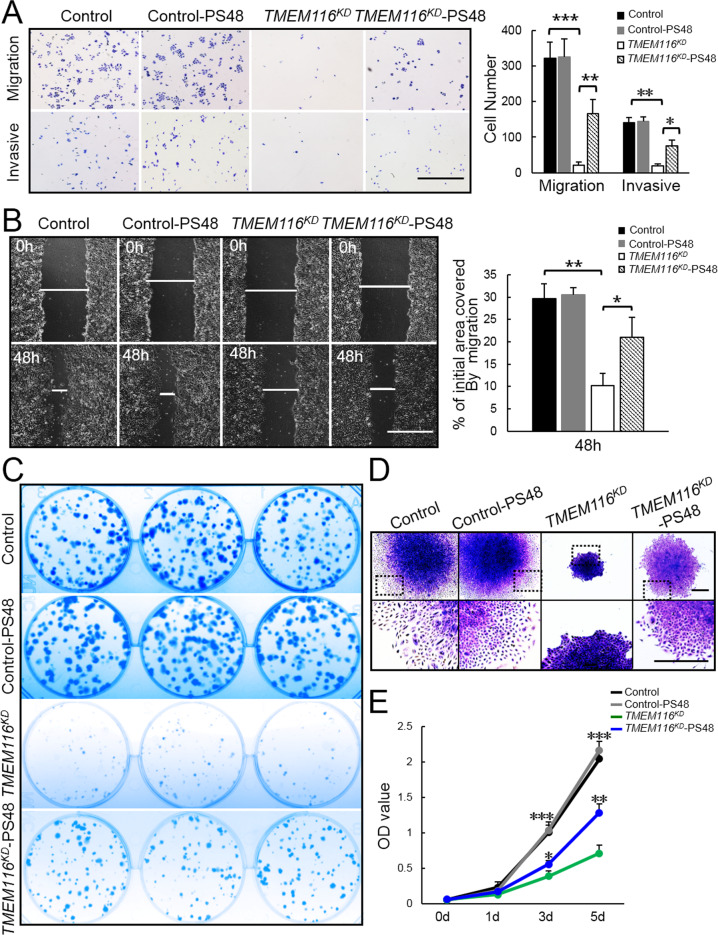
Activation of PDK1 largely restores the proliferation, clone formation, migration and invasion of *TMEM116*^*KD*^ cells. **A** Control, control-PS48, *TMEM116*^*KD*^ and *TMEM116*^*KD*^-PS48 cells were subjected to Transwell migration and invasion assays. More than three fields of cells in the lower chambers were counted. Scale bar: 1000 μm. **B** Control, control-PS48, *TMEM116*^*KD*^ and *TMEM116*^*KD*^-PS48 cells were subjected to wound-healing assay. Scale bar: 1000 μm. Representative images from over 30 non-overlapping fields at each time point are shown. **C** Control, control-PS48, *TMEM116*^*KD*^ and *TMEM116*^*KD*^-PS48 cells were subjected to colony formation assay. **D** Control, control-PS48, *TMEM116*^*KD*^ and *TMEM116*^*KD*^-PS48 cells were subjected to colony morphology analyses. Scale bar: 500μm. **E** Control, control-PS48, *TMEM116*^*KD*^ and *TMEM116*^*KD*^-PS48 cells were subjected to CCK8 assays at 0, 1, 3, 5 days. The bars represent the mean ± SD. *P < 0.05, **P < 0.01, ***P < 0.001. *n* = 6 mice. Representative images from three independent experiments are shown above.

To investigate the role of PDK1 in TMEM116 regulation of cancer cell proliferation, clone formation, migration and invasion, PS48 treated *TMEM116*^*KD*^ A549 cells were analyzed. In the cell migration and invasion assay, PS48 treatment significantly rescued cell migration and invasion in *TMEM116*^*KD*^ cells (Fig. 6A, B). In addition, the colony formation capability (Fig. 6C, D) and cell proliferation (Fig. 6E) were also largely activated by PS48 treatment in *TMEM116*^*KD*^ cells. Thus, the effects of TMEM116 on cancer cell proliferation, clone formation, migration, and invasion are mainly mediated through PDK1.

SC79 is a unique specific AKT activator, and directly enhances phosphorylation of all AKT isoforms and increases AKT activity in multiple cell types [30]. To investigate whether activation of AKT also restore the effects of TMEM116 on cancer cells, SC79 was used to treat *TMEM116*^*KD*^ and control cells. As expected, SC79 increased levels of p-AKT and p-FOXO3A (Supplementary Fig. 8). Consequently, TMEM116-knockdown-induced E-cadherin and p63 expression was blocked in SC79 treated *TMEM116*^*KD*^ cells. In migration and invasion assays, SC79 treatment also partly reversed TMEM116-knockdown-induced inhibitions of migration and invasion (Supplementary Fig. 9A, B). However, SC79 treatment failed to rescue colony formation capability and proliferation of the *TMEM116*^*KD*^ cells (Supplementary Fig. 9C–E). These results demonstrate that TMEM116 promotes cell migration and invasion, but not proliferation, partly through AKT/FOXO3A/TAp63 pathways.

## Discussion

**Fig. 7:**
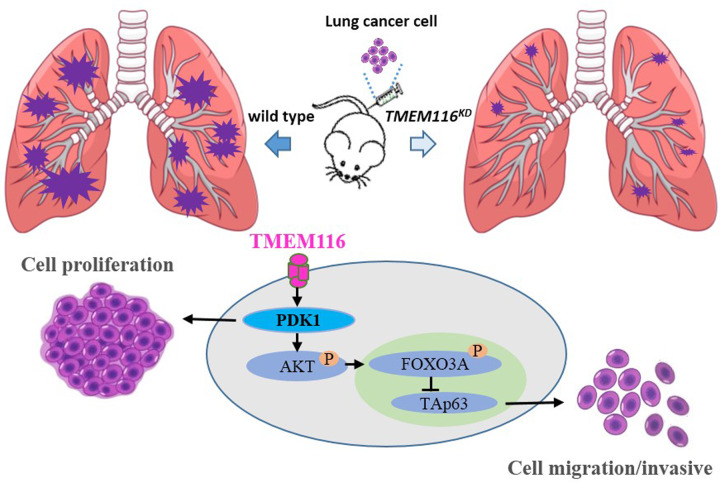
A working model that TMEM116 oncogenic signaling in cell motility and tumor metastasis. TMEM116 is required for lung cancer cell growth, motility and invasion. PDK1 signaling via AKT/FOXO3A/TAp63 is thought to be involved in this progression.

Membrane proteins constitute approximately 30% of the proteome [31]. Most of them play a fundamental role during cancer development or cancer cell dissemination notably by transmitting information between the extracellular environment and the cytoplasmic proteins [1]. Moreover, because of their large number and various biological functions, membrane proteins account for approximatively 60% of the targets of marketed drugs [32, 33]. Many transmembrane proteins remain poorly understood in term of their structures and functions and are simply grouped in the transmembrane protein (TMEM) family. However, most of them appear crucial for the metastatic cascade in several cancer types and their expression is often correlated with a poor prognosis for patient survival. In this study, we identify that TMEM116 is activated in human lung cancer clinical samples and mouse lung cancer model. TMEM116 knockdown in A549, and H1299 cells (Supplementary Fig. 10), showed that TMEM116 is required for lung cancer cell growth, motility and invasion. In consistent with in vitro data, TMEM knockdown dramatically reduced number of metastatic nodules formed in nude mice bearing *TMEM116*^*KD*^ cells comparing with mice injected with control A549 cells. Moreover, deficiency of TMEM116 in A549 cells significantly inhibits PDK1, phosphorylation of AKT and FOXO3A, and activates TAp63 expression, which in turn obstructs cell growth, migration, invasion, and metastasis. Restoration of PDK1 by PS48 treatment is able to largely recover the phosphorylation of AKT and obstruct expression of TAp63, and restore the cell growth, migration and invasion in *TMEM116*^*KD*^ cells. However, restoration of AKT^-P^ by SC79 treatment only partly reverse the expression of TAp63 and cell migration and invasion, but not cell growth in *TMEM116*^*KD*^ cells. It is plausible that other proteins/effectors might be responsible for PDK1 signaling on cell growth (Fig. 7).

Moving distally from the trachea, lung cancers show typical characteristics of squamous cell carcinoma, small-cell lung cancer and adenocarcinoma. This is mainly because compositions of lung cells change from the proximal to distal parts of the respiratory tract [8]. Although cellular heterogeneity in lungs defines diversity in the types of cancers, the precise cells where cancers originate are still poorly understand. In current study, we found that BaP-induced cancer cells and clinical tumor samples exhibit increased expression of TMEM116, whereas the epithelial markers are lost. These data support that the cellular function of epithelial cells is irreversibly changed during oncogenesis. Although it remains unclear whether aberrant increase of TMEM116 is the major cause or the consequence of the disease, alteration of TMEM116 might define a predictive biomarker of lung cancer, and help to develop diagnostic tools. Interestingly, by generating TMEM116-overexpression A549 cells, we found that the effects of TMEM116 on cell migration and invasion are not dose dependent. Since the expression of TMEM116 in cancer cells is already much higher than in normal epithelial cells, extra TMEM116 may not make differences (Supplementary Fig. 11). Tumors can be defined as a loss of tissue organization and aberrant behavior of cells that, growing independently and leaving primary sites to form metastatic colonies in surrounding tissue or distant organs. This process is largely responsible for cancer-associated mortality and epithelial-to-mesenchymal transition (EMT) [34, 35]. Our observation that mesenchymal markers were not detectable in the TMEM116^positive^ cancer cells suggests that TMEM116 may be essential for tumor cell migration/metastasis, but not EMT for primary cancer cells. It is also possible that the BaP-induced tumors are in early-stage, when the neoplastic cells remain in epithelial-like state. Whether TMEM116 signaling is involved in EMT in later stage of cancer development (malignant cancer cells) needs to be further investigated.

PI3K/AKT/mTOR pathway plays many important roles in tumor cell proliferation, survival, differentiation, invasion, migration, and metastasis [36–38]. PDK1 is downstream target of PI3K and mediates the phosphorylation of AKT [27]. Meanwhile, the mechanistic target of mTOR is known to regulate downstream signaling cascades by integrating both intracellular and extracellular signals [39]. Although PI3K/AKT and mTOR are two separate pathways, they are often considered as PI3K/mTOR signaling network owing to functional interconnectedness. Interestingly, in current study, we find that the deficiency of TMEM116 in A549 cells does not alter the level of PI3K and mTOR, but PDK1 and phosphorylation of AKT. This indicates that TMEM116 activation of PDK1/AKT signaling may be through unknown mediator(s), independent of PI3K and mTOR. Consistent with our data, it has been proposed that tumors with mutations in PI3K, but resistant to treatment with PI3K inhibitors, could be treated with PDK1 inhibitors. Moreover, several reports show that PDK1 is implicated in Ras/MAPK and Myc signaling pathways, which are frequently altered in cancer [40, 41]. Above findings suggest that PDK1 could regulate tumorigenesis in a PI3K independent manner [38]. By STRING (http://string-db.org/) analysis, we found no interaction occurred between TMEM116 and PDK1 protein (Supplementary Fig. 12). How TMEM116 regulates PDK1 signaling remains to be further investigated.

The PDK1 signaling pathway is complex. It, traditionally known to phosphorylate AKT, has been implicated in several signaling pathways altered in cancer. Indeed, PDK1 is responsible for the phosphorylation of many other AGC kinases, such as p70 ribosomal protein S6 kinase (p70S6K), serum/glucocorticoid regulated kinase (SGK), p90 ribosomal protein S6 kinase (p90RSK) and the members of protein kinase C (PKC) family [15]. There are convincing data demonstrating that increased PDK1 promotes tumor invasiveness and metastasis, while phosphorylation of AKT remains unaltered [39]. It is therefore not surprising that the restoration of AKT is unable to reverse the effects of PDK1 deficiency on *TMEM116*^*KD*^ cell proliferation. It is plausible that other protein may mediate the PDK1 effects on proliferation.

The *p63* gene is a member of the p53/p63/p73 family of transcription factors and plays a critical role in development and homeostasis of squamous epithelium. It includes two subclasses of proteins containing either TA or ΔN domains at the amino terminus [42]. Accumulating evidence indicates that the inactivation of TAp63 and ΔNp63 play a causative role in promoting cell motility and cancer metastasis [43, 44]. It has been reported that AKT/FOXO3A signaling pathway is responsible for p110α^H1047R^-, K-Ras^G12V^-, H-Ras^G12V^-, and Her2-mediated suppression of ΔNp63α expression and promotion of cancer cell motility and tumor metastasis [13]. In current study, we show that the lack of TMEM116 stimulates TAp63 expression through PDK1 pathway and in turn inhibits cancer cell motility and tumor metastasis. It is noteworthy that TAp63 and ΔNp63 shares common upstream effectors in regulating cancer cell motility and tumor metastasis. However, due to the lack of expression of ΔNp63 in A549 cells, whether TMEM116/PDK1 signaling pathway has effects on ΔNp63 expression in cancer development is query that has not been hitherto addressed.

In sum, the results of this study demonstrate that TMEM116 via PDK1/AKT/FOXO3A signaling pathway targets TAp63, which is critical in cancer cell motility and tumor metastasis. The activity of TMEM116 signaling pathway suggests a potential target to develop new strategy for the diagnosis and treatment of lung cancer.

## Supplementary information

supplemental figures

Author confirmations
