# Supplementary material for: TMEM116 is required for lung cancer cell motility and metastasis through PDK1 signaling pathway
Source: Cell Death Dis. 2021 Nov 16;12(12):1086. doi: 10.1038/s41419-021-04369-1 (PMC8599864; doi:10.1038/s41419-021-04369-1)
Supplement: Supplementary file 1 — supplemental figures [file 41419_2021_4369_MOESM1_ESM.docx]

**
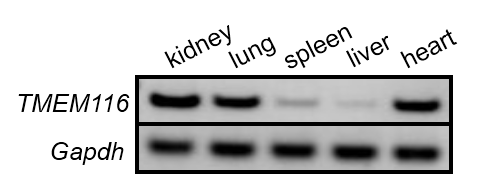
**

**Supplemental Figure 1. The expression pattern of *Tmem116* in mouse.**

The mRNA expression level of TMEM116 in mouse heart, liver, spleen, lung, and kidney were examined by RT-PCR analysis.

**
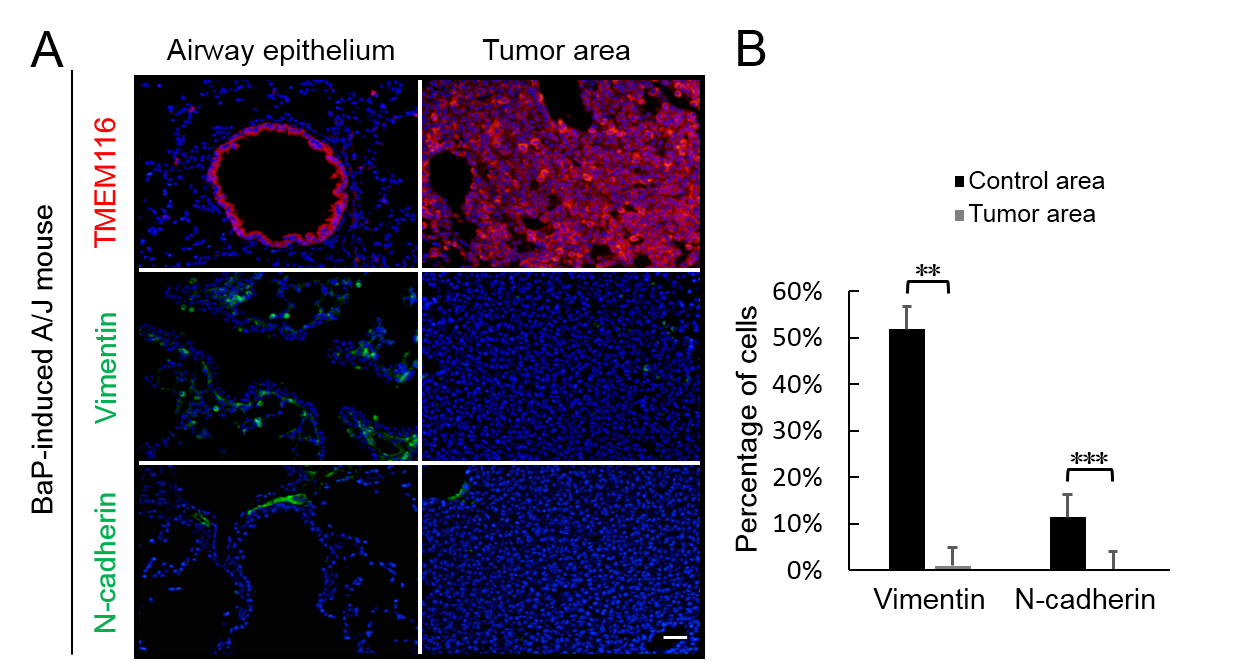
**

**Supplemental Figure 2. Detection of EMT in BaP-induced mouse lung cancer model.**

**A.** IF staining analysis of TMEM116, Vimentin and N-CAD expression in BaP-induced lung cancer tissue. Scale bar: 50μm. **B.** Quantification of Vimentin and N-cadherin positive cells. Percentages of Vimentin and N-cadherin positive cells in total cells were counted on control and tumor area. The bars represent the mean ± SD. ***P*< 0.01 ****P*<0.001.

**
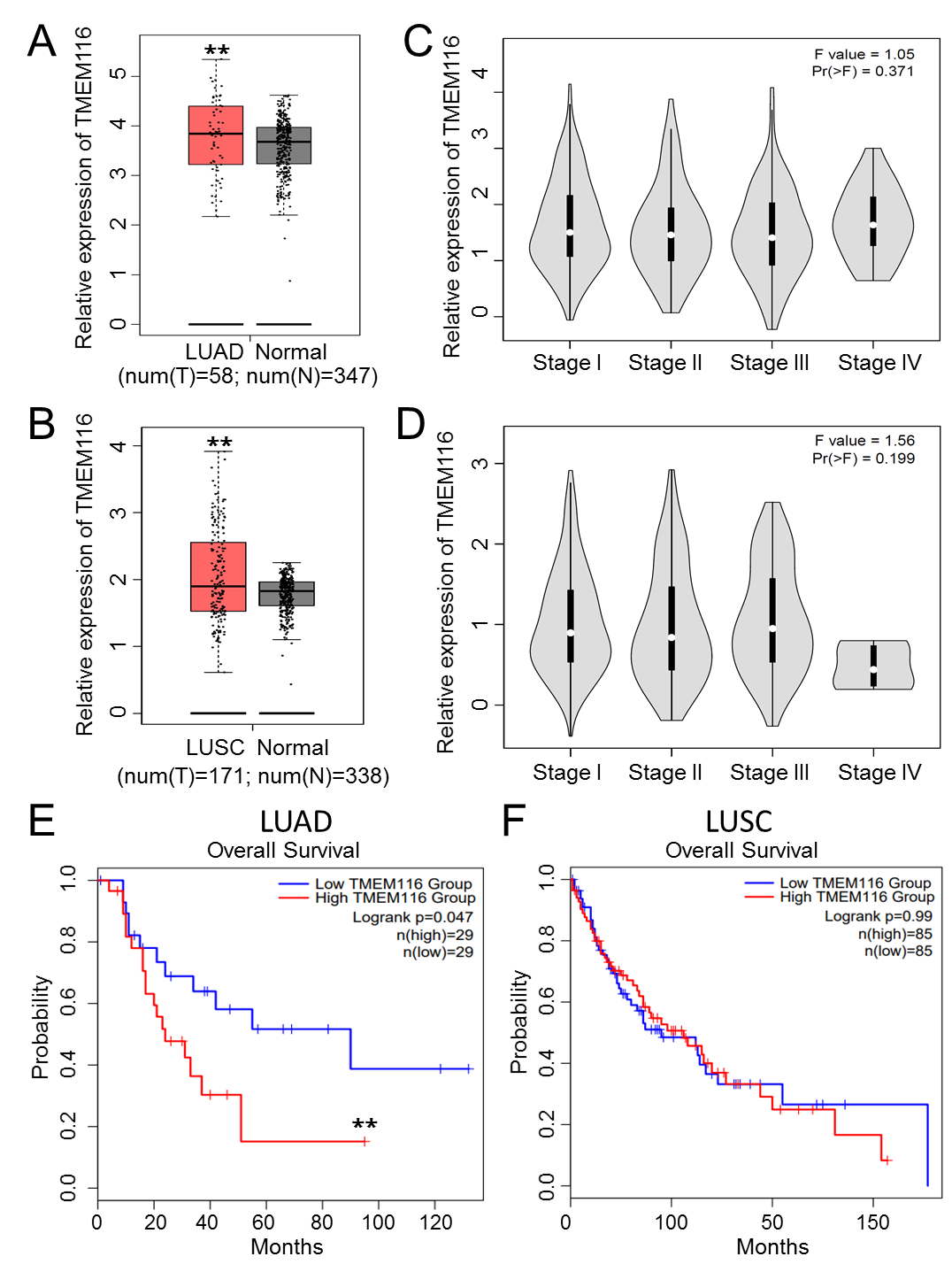
**

**Supplemental Figure 3. TMEM116 is upregulated and associated with shorter OS time in NSCLC.**

**A-B**. Expression of TMEM116 in LUAD and LUSC samples. **C-D**. Expression of TMEM116 in different stage of LUAD and LUSC samples. **E-F**. Kaplan-Meier Plotter analysis showed the correlation between TMEM116 expression and OS time in patients with LUAD (**E**) and LUSC (**F**). Significance was defined as ** *P* < 0.05.

**

**

**Supplemental Figure 4. Generation of TMEM116-knockdown A549 cell line.**

**A.** The sequence of sgRNA-TMEM116 designed by Guide Design Resources. **B.** The genotyping of *TMEM116^KD^* A549 cell line by DNA sequencing. **C.** TMEM116 expression in *TMEM116^KD^* and control A549 cells was subjected by Western blotting analyses. The bars represent the mean ± SD. ***P*< 0.01.


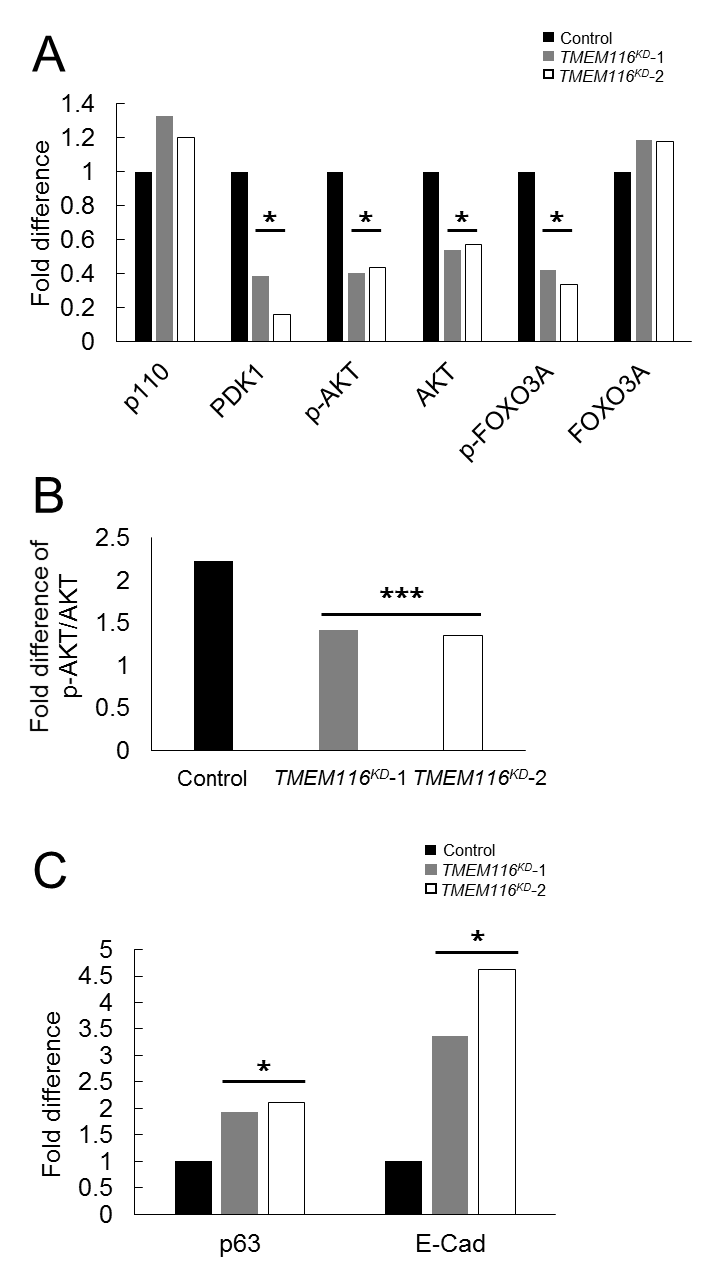


**Supplemental Figure 5. Quantifications of western blot analysis.**

**A.** The western blot analysis of TMEM116 pathway related genes in control and *TMEM116^KD^* cells were quantified by Image J. **B.** The analysis of p-AKT/AKT ratio in control and *TMEM116^KD^* A549 cells. C. The western blot analysis of p63 and E-cadherin 1 in control and *TMEM116^KD^* cells were quantified by Image J. **P*< 0.05, ****P*< 0.001. , and PS48 treated


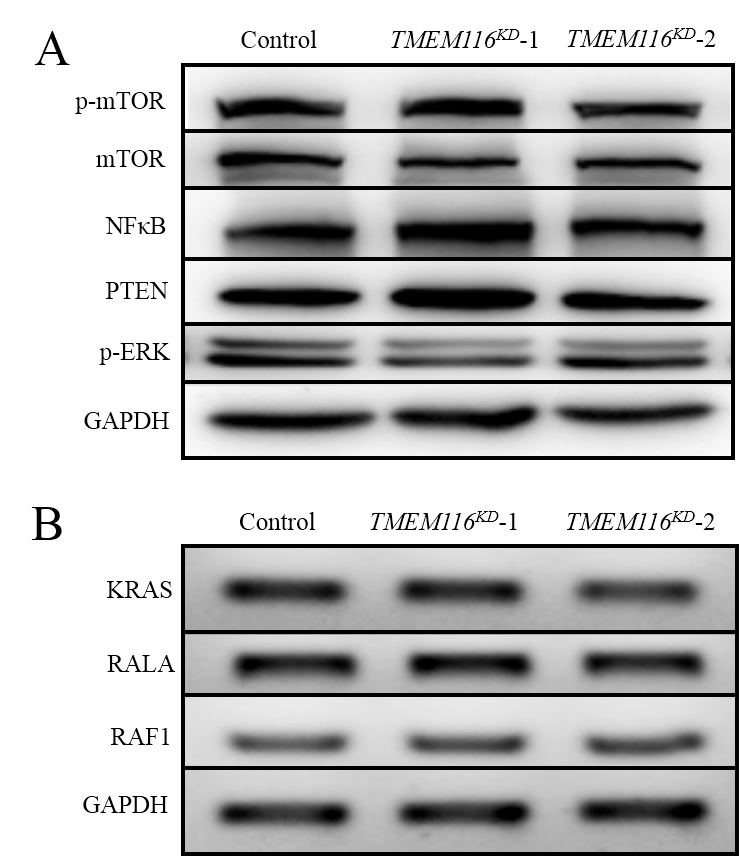


**Supplemental Figure 6. Expression of oncogenesis related genes are analyzed by western blot and RT-PCR.**

**A.** p-mTOR, mTOR, NFκB, PTEN, and p-ERK expression in *TMEM116^KD^* and control cells was determined by western blot. **B.** *KRAS, RALA,* and *RAF1* expression in *TMEM116^KD^* and control cells was analyzed by RT-PCR.


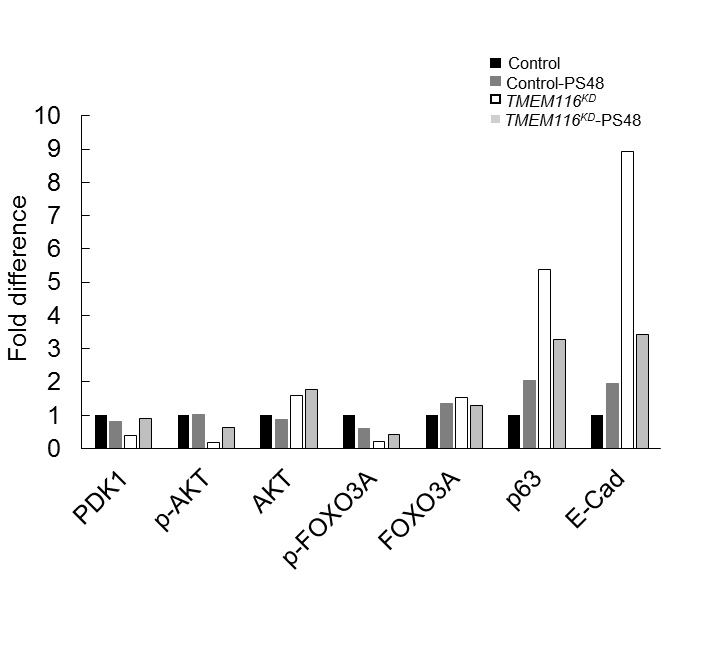


**Supplemental Figure 7. Quantifications of western blot analysis.**

The western blot analysis of TMEM116 pathway related genes in control, *TMEM116^KD^*, and PS48 treated cells were quantified by Image J. **P*< 0.05, ****P*< 0.001.


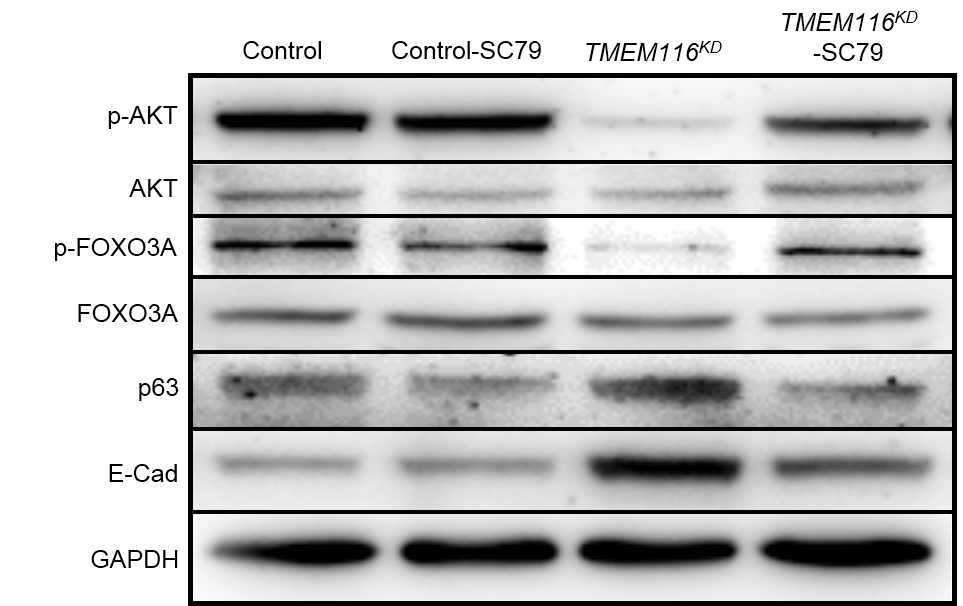


**Supplemental Figure 8. AKT activation by SC79 partially restores AKT/FOXO3a/TAp63 signaling pathways.**

P-AKT, AKT, p-FOXO3A, FOXO3A, TP63, and E-cadherin expression in Control, Control-SC79, *TMEM116^KD^* and *TMEM116^KD^* -SC79 cells was determined by western blot.

**
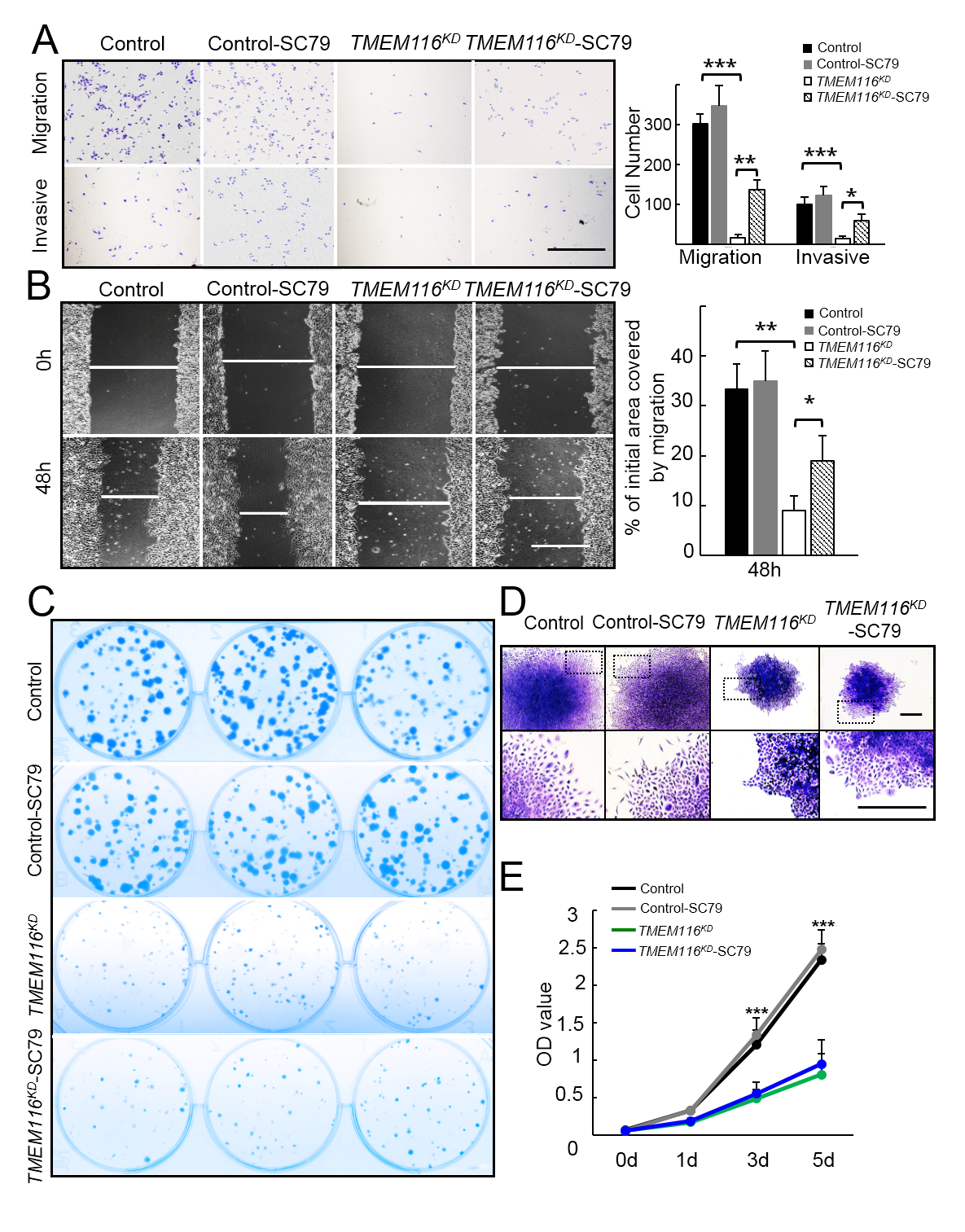
**

**Supplemental Figure 9. Activation of AKT by SC79 partially restores the migration and invasion of TMEM116 knockdown cells.**

**A.** Control, Control-SC79, *TMEM116^KD^* and *TMEM116^KD^*-SC79 cells were subjected to Transwell migration and invasion analyses. Scale bar: 1000μm. **B.** Control, Control-SC79, *TMEM116^KD^* and *TMEM116^KD^*-SC79 cells were subjected to wound healing analyses. Scale bar: 1000μm. **C.** Control, Control-SC79, *TMEM116^KD^* and *TMEM116^KD^*-SC79 cells were subjected to colony formation analyses. **D.** Control, Control-SC79, *TMEM116^KD^* and *TMEM116^KD^*-SC79 cells were subjected to colony morphology analyses. Scale bar: 500μm. **E.** Control, Control-SC79, *TMEM116^KD^* and *TMEM116^KD^*-SC79 cells were subjected to CCK8 assays at 0, 1, 3, 5, 7 days. The bars represent the mean ± SD. **P*< 0.05, ***P*< 0.01, ****P*< 0.001.


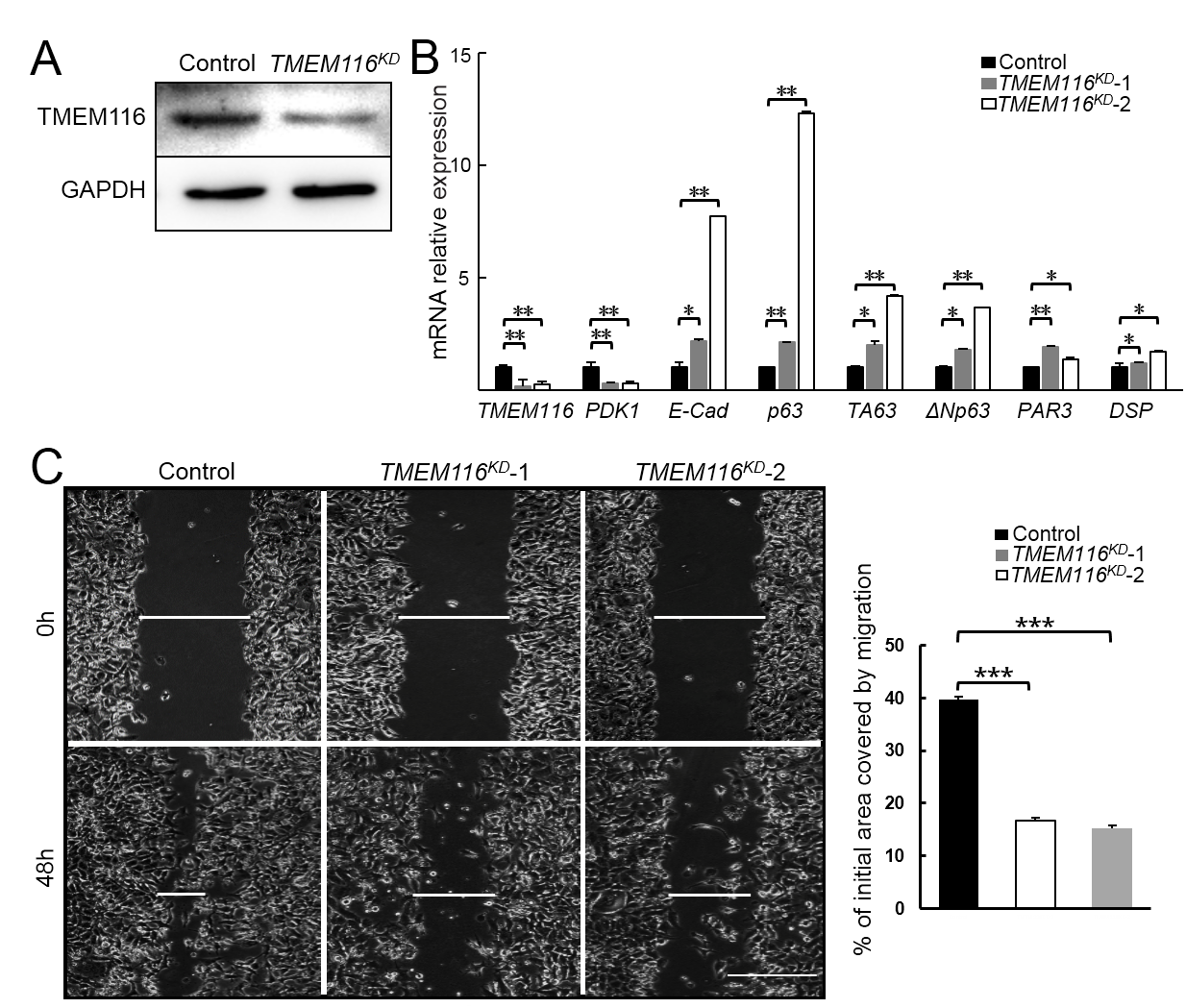


**Supplemental Figure 10. TMEM116 deficiency in H1299 cells inhibits PDK1 signaling and cell migration.**

**A.** Western blot analysis of TMEM116 expression in *TMEM116^KD^* H1299 cells. **B.** By real-time PCR analysis, the expression of PDK1 was down-regulated in *TMEM116^KD^* H1299 cells. **C.** Control, *TMEM116^KD^*-1 and *TMEM116^KD^*-2 H1299 cells were subjected to wound healing analyses. Scale bar: 500μm.


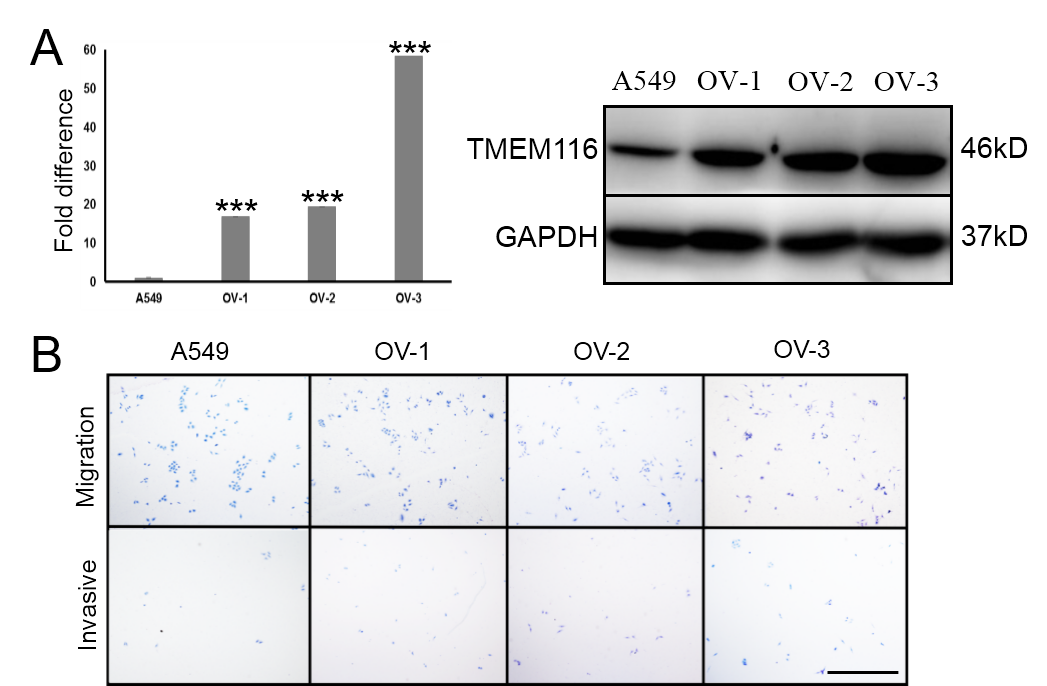


**Supplemental Figure 11. Generation of *TMEM116*-overexpression A549 cell line.**

**A.** The expression of *TMEM116* in A549 and three *TMEM116*-overexpression cell lines was examined by real-time PCR and western blot. **B.** A549 and *TMEM116*-overexpression cells were subjected to Transwell migration and invasion analyses. Scale bar: 1000μm.

**
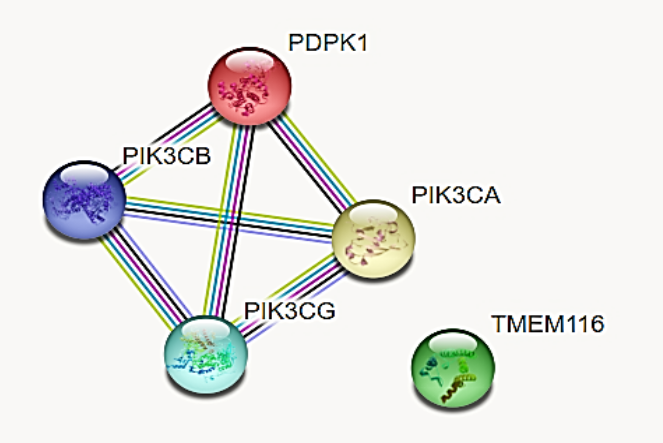
**

**Supplemental Figure 12. TEME116 protein does not interact with PDK1.**

By STRING analysis, there is no interaction between TMEM116 protein and PDK1 protein.

**

**

**Supplemental Table 1: Primer list.**

The primers used in this study were listed.

**

**

**Supplemental Table 2: Primary antibody list.**

The primary antibodies used in this study were listed.
