# Supplementary material for: TMEM116 is required for lung cancer cell motility and metastasis through PDK1 signaling pathway
Source: Cell Death Dis. 2021 Nov 16;12(12):1086. doi: 10.1038/s41419-021-04369-1 (PMC8599864; doi:10.1038/s41419-021-04369-1)
Supplement: Supplementary file 2 — Author confirmations [file 41419_2021_4369_MOESM2_ESM.pdf]

Dear Dr. Xing,

I am writing to confirm that I give my consent for information about myself to be published in Cell Death & Disease, CDDIS-21-2663RR, "TMEM116 is required for lung cancer cell motility and metastasis through PDK1 signaling pathway".

Sincerely

Dr. Suhong Zhang

Suhong Zhang

State Key Laboratory for Agrobiotechnology
College of Biological Sciences
China Agricultural University
Beijing, P.R. China.
Email: SZ20143020066@cau.edu.cn

Dear Dr. Xing,

I am writing to confirm that I give my consent for information about myself to be published in Cell Death & Disease, CDDIS-21-2663RR, “TMEM116 is required for lung cancer cell motility and metastasis through PDK1 signaling pathway”.

Sincerely

Dr. Haiting Dai

State Key Laboratory for Agrobiotechnology
College of Biological Sciences
China Agricultural University
Beijing, P.R. China.
Email: SZ20173020178@cau.edu.cn

Dear Dr. Xing,

I am writing to confirm that I give my consent for information about myself to be published in Cell Death & Disease, CDDIS-21-2663RR, “TMEM116 is required for lung cancer cell motility and metastasis through PDK1 signaling pathway”.

Sincerely

Dr. Wenya Li

State Key Laboratory for Agrobiotechnology
College of Biological Sciences
China Agricultural University
Beijing, P.R. China.
Email: b20203020120@cau.edu.cn

Dear Dr. Xing,

I am writing to confirm that I give my consent for information about myself to be published in Cell Death & Disease, CDDIS-21-2663RR, “TMEM116 is required for lung cancer cell motility and metastasis through PDK1 signaling pathway”.

Sincerely

Dr. Runming Wang
State Key Laboratory for Agrobiotechnology
College of Biological Sciences
China Agricultural University
Beijing, P.R. China.
Email: wrm1990@cau.edu.cn

Dear Dr. Xing,

I am writing to confirm that I give my consent for information about myself to be published in Cell Death & Disease, CDDIS-21-2663RR, “TMEM116 is required for lung cancer cell motility and metastasis through PDK1 signaling pathway”.

Sincerely

Dr. Hanyu Wu

Hanyu Wu.

State Key Laboratory for Agrobiotechnology
College of Biological Sciences
China Agricultural University
Beijing, P.R. China.
Email: wuhanyu@cau.edu.cn

Confirmation letter from Dr. Ming Shen

锁屏 退出 邮件全文搜索

收件箱 署名同意信 ×

回复 回复全部 转发 移动到 标记为 更多 删除

署名同意信

沈鸣 发送给 邢怡明

发起会议

2021-10-20 13:03:35

Dear Dr. Xing,

I am writing to confirm that I give my consent for information about myself to be published in Cell Death & Disease, CDDIS-21-2663RR, "TMEM116 is required for lung cancer cell motility and metastasis through PDK1 signaling pathway".

Sincerely

Shen Ming
Translational Medicine Center,
Chinese PLA General Hospital(301 hospital),
No. 28 Fu Xing Road (Wukesong),
Haidian District, Beijing 100853, China
Office: 86-10-66936362
Cell phone: 86-15001342876

Dear Dr. Xing,

I am writing to confirm that I give my consent for information about myself to be published in Cell Death & Disease, CDDIS-21-2663RR, "TMEM116 is required for lung cancer cell motility and metastasis through PDK1 signaling pathway".

Sincerely

Dr. Ye Hu

Ye Hu

College of Pulmonary and Critical Care Medicine
Chinese PLA General Hospital
Beijing, P.R.China.

Dear Dr. Xing,

I am writing to confirm that I give my consent for information about myself to be published in Cell Death & Disease, CDDIS-21-2663RR, “TMEM116 is required for lung cancer cell motility and metastasis through PDK1 signaling pathway”.

Sincerely

Prof. Lixin XIE

Head of College of Pulmonary & Critical Care Medicine
Chinese PLA General Hospital
Beijing
China
